# A Study of Knowledge, Attitudes and Practices Relating to Brucellosis among Small-Scale Dairy Farmers in an Urban and Peri-Urban Area of Tajikistan

**DOI:** 10.1371/journal.pone.0117318

**Published:** 2015-02-10

**Authors:** Elisabeth Lindahl, Nosirjon Sattorov, Sofia Boqvist, Ulf Magnusson

**Affiliations:** 1 Division of Reproduction, Department of Clinical Sciences, Swedish University of Agricultural Sciences, P.O. Box 7054, SE- 750 07 Uppsala, Sweden; 2 Tajik Agrarian University, P.O. 734003 Dushanbe, Tajikistan; 3 Division of Food safety and Bacteriology, Department of Biomedical Sciences and Veterinary Public Health, Swedish University of Agricultural Sciences, P.O. Box 7028, SE- 750 07 Uppsala, Sweden; Curtin University, AUSTRALIA

## Abstract

Improvement of knowledge, attitudes and practices among urban livestock farmers could have a significant impact on the reduction of many zoonotic infections in urban farming. This study aimed to describe and evaluate weak areas in knowledge, attitudes and practices with regards to brucellosis among urban and peri-urban small-scale dairy farmers in a low income country to generate information essential for control programmes and public health interventions. The cross-sectional study was conducted during six weeks in 2011. The study subjects were small-scale dairy farmers living in the urban and peri-urban area of the capital Dushanbe in Tajikistan. In total, 441 farmers were interviewed using a questionnaire with questions about demographic characteristics, knowledge, attitudes and practices relating to brucellosis. Descriptive statistics were used and a logistic regression model applied to evaluate potential predictors to knowledge about brucellosis. The majority (85%) of the farmers had never heard of brucellosis. Low educational level was found to be associated with low awareness of brucellosis (P = < 0.001). Respondents who talked about animal health issues with family members or friends were less likely to have heard of brucellosis compared to those who often talked to veterinarians (P = 0.03). Sixty three per cent of the participants wanted more information about brucellosis. Seventeen per cent sold unpasteurized dairy products on a regular basis direct to consumers. Almost 30% of the households consumed unpasteurized dairy products on regular basis. A majority of the respondents did not use any protection when handling cows having an abortion or when dealing with aborted materials. Poor knowledge, high-risk behaviours and a willingness to learn more strengthens the logic for including health education as part of control programmes.

## Introduction

Brucellosis, caused by *Brucella* species, is considered a neglected zoonosis by the World Health Organisation (WHO) [1]. The disease is widespread in many low-income countries and although low mortality rate in humans, infection might develop into a disabling chronic illness with osteoarticular manifestation being a common complication [2–3]. In many low-income countries including Tajikistan the incidence of human and animal brucellosis is increasing [3] and lack of awarness, policies or appropriate use of resources are contributing factors to this development [4]. A study from the neighbouring country Kyrgyzstan indicates an under-reporting of human brucellosis cases [5]. The most common transmission routes of *Brucella* infection in humans are through direct contact with infected livestock or consumption of unpasteurized dairy products [6–7]. In livestock, it causes reproductive disorders and a decrease in milk production. Transmission between livestock occurs mainly due to the large amounts of bacteria shed in the birth fluids when an infected female aborts or give birth [8–9].

Urban and peri-urban farming is a common practice in Tajikistan and other low-income countries [10–11]. This practice is an opportunity for the dairy farmers to improve their livelihood but might also pose a threat for animal and public health if zoonotic pathogens like *Brucella* spp. are present. Notably, a seroprevalence study conducted in 2011 showed that four per cent of the urban and peri-urban located dairy herds around the capital Dushanbe were infected with brucellosis [12]. Improvement of knowledge, attitudes and practices (KAP) among urban livestock farmers could have an important impact on the reduction of many zoonotic infections in urban agriculture [13].

In order for a control programme to be efficient, it is important with a good understanding of local knowledge, attitudes and practices relating to brucellosis to improve information delivery and initiate relevant control measures. It might also be advantageously for the outcome of a control programme to include disease education among community participants [4]. A KAP study of brucellosis conducted in Kenya among people with high level of contact with livestock implied that the disease awareness and knowledge of the transmission routes were poor [14]. Another KAP study conducted in Egypt showed a relative high general knowledge of brucellosis but still a high-risk behavior among livestock owners which the authors concluded might contribute to a high seroprevalence of brucellosis among large ruminants in the area [15]. The objective of the current study was to describe and evaluate weak areas in knowledge, attitudes and practices among dairy farmers with regards to brucellosis in urban and peri-urban small-scale farming in a Central Asian low-income country. Such information is essential for future control programmes and public health interventions.

## Materials and Methods

### Study area

The study subjects were small scale dairy farms in the urban and peri-urban area of the capital Dushanbe populated by approximately 700 000 [16]. This region has a high density of people and cattle living together and is dominated by small-scale dairy farming with approximately one to three cows per household (N. Sattorov 2013, personal communication).

### Study design

There were 441 households in 32 villages enrolled in this cross-sectional study. The original data set contained 443 households, but two were omitted due to missing data. The selection of households has been described previous in a *Brucella* seroprevalence study conducted by Lindahl et al [12]. In brief, villages in the study area were given numbers on small cards and selected randomly from a bowl without replacement. The selection of households within each village was based on if the family was at home, the possession of dairy cows and on the willingness of the farmer to participate in the study. One village was visited per day and as many households as possible were interviewed. As the current study was conducted simultaneously with a seroprevalence study [12] one village had to be excluded due to all the cows were let out on communal grazing before the arrival of the study team. The closest nearby village was selected as replacement. Two farmers refused to participate and three farmers were not at home and therefore unable to participate.

### Study procedure

A questionnaire with approximately 50 questions on demographic characteristics, knowledge, attitudes and practices relating to brucellosis was developed by the authors and pre-tested to allow for improvements. The questions were either open-ended or dichotomous. All interviews were performed orally at visits in the households by the authors N.S and E.L during three weeks in May and three weeks in October 2011. The family member responsible for the daily management of the cows was interviewed in Tajik, Russian or Uzbek depending on the person´s native language.

### Ethics statement

All participants were informed about the purpose and methods of the study, that the data would be handled anonymously and that participation was on voluntary basis. Informed verbal consent was obtained from all participants and documented in the questionnaire. The current study was conducted simultaneously with a *Brucella* seroprevalence study among dairy cows [12]. No data regarding the identity of the farmers were collected in order to make sure that information obtained from the seroprevalence study could not be traced back to the individual farmer. This was important because the farmers would not receive any financial compensation if a cow was found to be infected with *Brucella* and hence at risk of being culled. Therefore, collecting any data regarding identity would risk many farmers to refuse to participate in the study. The study and verbal consent procedure was approved by the University ethical committee at the Tajik Agrarian University (Dushanbe, Tajikistan) chaired by the Rector. The study was conducted according to the ethical standards of Tajik Agrarian University and in correspondence with the Swedish legislation of ethics in research involving humans [17].

### Statistical analysis

Data from the questionnaires were entered in Excel software (Microsoft) and statistical analyses conducted in SAS version 9.3 (Cary NC, USA). Descriptive statistics were used for demographic characteristics and knowledge relating to brucellosis. For investigating potential predictors to knowledge about brucellosis, i.e. if the farmer had heard about the disease, univariable logistic regression analyses were used. Investigated explanatory variables were gender, level of education, number of people in the household (defined as family members regularly sharing meals), native language and who the farmer discussed animal health issues with. Due to its small size, a group of three illiterate respondents within the group level of education had to be removed from the data in order to perform the analyses resulting in 438 included households. Variables with a P-value < 0.2 were entered in a multivariable logistic regression model with manual backward elimination until all remaining variables showed a P ≤ 0.05. The variable “number of people in the household” had a P-value > 0.2 and was therefore excluded from the multivariable model. The statistical significance level was defined as a two-tailed P-value ≤ 0.05. The model was investigated for interactions and confounding. Confounding was investigated by adding the eliminated variables in the final model. A variable was considered to be a confounder if it changed the coefficient of the significant variables by >25%. The fit of the model was assessed using Hosmer and Lemeshow goodness-of-fit test. Descriptive statistics were applied for questions on attitudes and practices and univariable logistic regression was used to investigate association between a history of reported *Brucella* infection in humans, cattle, sheep or goats within the household and the practice of drinking or selling unpasteurized dairy products.

## Results

### Demographic characteristics

In the majority (78%) of the 441 included households, a female was main responsible for the management of the cows and most households were populated by six to ten people (Table 1). Eighty five per cent of the respondents had completed secondary school and less than one per cent was illiterate.

**Table 1:** Demographic characteristics of cattle farmers in the urban and peri-urban area of Dushanbe, Tajikistan (n = 441).

	**Category**	**n**	**%**
Gender responsible for taking care of the cows	Female	342	78
	Male	99	22
Native language	Tajik	368	83
	Uzbek	72	16
	Russian	1	0.2
Nr of people in the household^a^	1–5	62	14
	6–10	265	60
	>10	114	26
Level of education	Illiterate	3	0.7
	Primary	6	1.4
	Secondary	376	85
	Technical	20	4.5
	University	36	8.2

^a^Household defined as family members regularly sharing meals.

### Knowledge of brucellosis

The majority (85%) of the 441 respondents had never heard of brucellosis. Of those who had heard of the disease (n = 65), about half (n = 36) had received information from relatives or friends and the majority (n = 53) knew that cattle, sheep or goats could become infected (Table 2). All interviewees who had heard of brucellosis knew that humans could become infected and 52 persons responded that arthritis was a common symptom in humans. A majority (n = 51) did not know that cattle could be vaccinated against the disease. The majority (n = 59) of those who had heard of brucellosis knew at least one correct route of transmission from animals to humans, most commonly consumption of unpasteurized milk from infected cows. Fewer (n = 14) knew one or more correct route of transmission between animals. In five of the households, which had heard of brucellosis, a family member had been diagnosed with the disease by a physician and in two of the households a veterinarian had diagnosed brucellosis among cattle, sheep or goats.

**Table 2:** Knowledge about brucellosis among the respondents who had heard of the disease in the urban and peri-urban area of Dushanbe, Tajikistan (n = 65).

	**Category**	**n**	**%**
Information source	Relative/friends	36	55
	Veterinarian	14	22
	Book	7	11
	Television	1	1.5
	Don´t know	7	11
Which animal species can become infected	Cattle/Sheep/Goat	53	82
	All mammals	3	4.6
	Don´t know	9	14
Can humans become infected	Yes	65	100
	No	0	0
Symptoms in humans	Arthritis	52	80
	Fever and arthritis	2	3.1
	Fatigue	1	1.5
	Skin lesions	2	3.1
	Don´t know	8	12
Exist any vaccination for animals	Yes	14	22
	No	51	78
Modes of transmission:			
Animal-to-animal	Correct^a^	14	22
	Non correct^b^	51	78
Animal-to-human	Correct^a^	59	91
	Non correct^b^	6	9.2
Previous *Brucella* infection within the household:			
Among humans	Yes	5	7.7
	No	60	92
Among cattle/sheep/goats	Yes	2	3.1
	No	63	97

^a^Stated at least one correct route of transmission.

^b^Stated no correct route of transmission.

The multivariable model showed that respondents with Tajik as native language were more likely to have heard of the disease compared to those having Uzbek or Russian as native language (P = 0.03) (Table 3). Participants with lower level of education were less likely to have knowledge of brucellosis compared to those who had attended technical college or university (P = < 0.001). Respondents who talked about animal health issues with family members or friends were less likely to have heard of brucellosis compared to those who often talked to veterinarians about animal health issues (P = 0.03). There was no difference in knowledge of brucellosis between men and women. No interactions or confounding were found in the multivariable model. The P-value was 0.4 for Hosmer and Lemeshow goodness-of-fit test, which indicates a good fit of the model.

**Table 3:** Results of multivariable logistic regression analysis investigating potential predictors to knowledge about brucellosis i.e. if the farmer had heard about the disease, among 438 households in Dushanbe, Tajikistan.

**Variable**	**Category**	**β**	**P**	**OR (95% CI)**
Native language	Tajik	1.1	0.03	2.9 (1.1–7.7)
	Uzbek/Russian			
Level of education	Primary/secondary	-1.2	< 0.001	0.3 (0.2–0.6)
	Technical/university			
Who the respondent talks to regarding animal health issues	Family member/friend	-0.6	0.03	0.5 (0.3–0.9)
	Veterinarian			

### Attitudes

Sixty three per cent (n = 279) of the households wanted more information about brucellosis whereas 37% claimed that they did not need more information. Of the 279 respondents who wanted more information, the majority (58%) preferred to receive the information through an educational booklet while 23% preferred a course or information meeting in the village. Eight per cent wanted information about brucellosis from a veterinarian and only one per cent from a television programme. Ten per cent of the respondents who wanted more information did not have any suggestion on how to best receive it.

Of the respondents who had heard of brucellosis (n = 65), only eleven believed some of their family member were at risk of contracting brucellosis and every one of those considered the person in the household working most with the cows exposed to the highest risk. The majority (n = 52) did not consider any family member at risk of contracting *Brucella* infection.

### Self-reported practices

Seventeen per cent (n = 76) of the respondents sold unpasteurized milk or unpasteurized milk products on a regular basis direct to consumers. Three of the respondents had to be removed from this question as it was uncertain whether they had understood the question correctly. The majority (66%) of these 76 respondents sold their unpasteurized dairy products on an everyday basis (Table 4). Close to 30% of the households consumed unpasteurized dairy products from the cows on regular basis. Females were more likely to assist during calving (56%) compared to males and it was almost as common to discuss animal health issues with veterinarians (48%) as it was with family members or friends (52%). Almost all households (94%) stated that they usually buried dead cattle fetuses. Seventy eight per cent washed their hands after dealing with cows having an abortion or with aborted materials whereas 21% used protection like gloves. When purchasing new cattle, the majority (63%) stated not taking any specific action to make sure the animal was healthy and 32% used more experienced people in the village for help. Most respondents (81%) declared that they contacted a veterinarian if any cattle showed symptoms of disease whereas 17% usually tried to treat the animal without consulting a veterinarian.

**Table 4:** Descriptive results of self-reported practices among dairy farmers in the urban and peri-urban area of Dushanbe, Tajikistan.

	**Category**	**n**	**%**
Does the respondent sell unpasteurized milk or unpasteurized milk products direct to consumers (n = 438)	Yes	76	17
	No	362	83
How frequent does the respondent sell unpasteurized milk or unpasteurized milk products (n = 76)	Every day	50	66
	One to two times per week	14	18
	Once a month/sometimes	12	16
Does the respondent consume unpasteurized milk or unpasteurized milk products (n = 441)	Yes	123	28
	No	318	72
Who in the household assist during calving (n = 441)	Female	246	56
	Male	138	31
	Female & Male	56	13
	Always call veterinarian	1	0.2
Who does the respondent talk to about animal health issues (n = 441)	Family member/friend	229	52
	Veterinarian	212	48
What does the respondent do with dead cattle fetuses (n = 441)	Bury	413	94
	Call veterinarian	9	2
	Feed for dog	7	1.6
	Burn	2	0.5
	Don´t know	10	2.3
Does the respondent use protection when dealing with cows having an abortion or with aborted materials (n = 441)	Use gloves	93	21
	Wash hands	344	78
	Always call veterinarian	1	0.2
	No / Don´t know	3	0.7
If the respondent buys a new cattle, does he/she take any action to assure it is healthy (n = 441)	No	280	63
	Use more experienced people in village	142	32
	Use veterinary inspection	19	4.3
What does the respondent do if a cattle is sick/ shows signs of disease (n = 441)	Seek veterinary assistance	359	81
	Treat	77	17
	Slaughter	4	0.9
	Don´t know	1	0.2

Univariable logistic regression analyses showed that households with a history of reported *Brucella* infection among humans, cattle, sheep or goats were equally inclined to sell and consume unpasteurized dairy products as those who had not had the infection within the household or who had never heard of the disease.

## Discussion

This study shows that the knowledge of brucellosis is poor among the dairy farmers in the urban and peri-urban area of the capital city in Tajikistan. Several known high-risk behaviors were common self-reported practices among the farmers. Such behaviors were consumption of unpasteurized dairy products and not wearing gloves when dealing with cows having an abortion or with aborted materials.

The majority of the respondents had never heard of the disease brucellosis. Similar results have been shown in a study from Kenya [14]. In contrast to this finding, a study in Uganda showed a high awareness of brucellosis among the community participants [18]. Similar results have been shown in Egypt where the majority of the farmers were aware of brucellosis which the authors explained by an endemic situation of brucellosis in the study area [15]. The low awareness of brucellosis in the current study might be explained by a lower herd seroprevalence among the dairy cows in the study area compared to Egypt [12]. However, it is noteworthy that the awareness of brucellosis was poor among the farmers despite a control programme among small ruminants initiated in 2003 by the Food and Agricultural Organisation (FAO) of the UN. The programme did not include the very region of Dushanbe but several areas in Tajikistan with high seroprevalences of *Brucella* spp. in sheep and goats [19–20]. A study from Kyrgyzstan showed that a good knowledge of the transmission routes for brucellosis had a protective effect for human infection [21] and a study from Iran that knowledge of the mode of brucellosis transmission by fresh cheese was protective against disease transmission in humans [22]. The human incidence of brucellosis is increasing in Tajikistan [3] and the majority of the farmers in the current study could be exposed to a higher risk of contracting *Brucella* infection due to a low awareness of the disease.

Participants with a lower level of education were less likely to have heard of brucellosis compared to those with a higher level of education but there was no difference in knowledge between men and women. The farmers with a lower level of education are thus likely at higher risk of contracting brucellosis. This is also supported by a study conducted in Yemen which showed that humans diagnosed with brucellosis were more likely to have a lower educational level compared to the controls [23].

The literacy rate among the study population was almost 100% and the majority of the respondents had completed secondary school which is in accordance with statistics from the UN database regarding Tajikistan [24]. More than half of the farmers wanted more information about brucellosis and the majority preferred to receive it through an educational booklet. The high literacy rate and educational standard, together with a positive attitude towards learning more, build a good foundation for including information campaigns for brucellosis as part of a future control programme in Tajikistan.

One third of the households consumed unpasteurized dairy products on regular basis. Consumption of unpasteurized dairy products is known to be an important risk factor for human brucellosis [7], [21–22], [25]. A fifth of the households also sold unpasteurized dairy products direct to consumers on regular basis. A seroprevalence study conducted among the dairy cows owned by the farmers who participated in the current KAP-study showed that the herd seroprevalence was approximately four per cent [12]. Hence, the practice of trading with unpasteurized dairy products could constitute a risk to public health. Changed political and economic situation has led to a privatization of collective farms in Tajikistan and other Central Asian countries [11], [21]. Kozukeev et al. suggest that this development has led to more frequent trading with home-made animal products in Kyrgyzstan, resulting in impaired food safety [21]. As the pattern of privatization of collective farms has been similar in Tajikistan as in Kyrgyzstan, there are reasons to believe that trading with Tajik home-made animal products has increased, putting food safety at risk.

A majority of the farmers did not use protective gloves when dealing with cows having an abortion or with aborted materials. One explanation for this could be poor knowledge of the risk with this practice but also lack of access to protective clothing like gloves. Similar results have been reported in a study from Egypt [15]. This practice is a known risk factor for humans [21], [25].

Notably, those households with a history of brucellosis in humans or livestock were equally inclined to trade with and consume unpasteurized dairy products as those who lived in a household with no history of brucellosis. This might imply that there is a lack of information from physicians and veterinarians to affected farmers regarding the modes of transmission of brucellosis.

It was almost as common to discuss animal health issues with veterinarians as it was with family members or friends. The majority of the farmers contacted a veterinarian if a cow showed symptoms of disease. This is in line with findings from a study conducted in Egypt where most respondents would contact the local veterinarian if they suspected brucellosis infection among their livestock [15]. In the current study it was shown that participants who mainly consulted veterinarians regarding animal health issues were more likely to have heard of brucellosis compared to those who mainly consulted a family member or a friend. The well-established relationship between many veterinarians and farmers could be useful if implementing information campaigns as part of a future control programme.

In the current study potential biases could have arisen if the questions were interpreted incorrectly by the farmers. However, the questionnaire was developed by the authors and N.S has a good insight in the small-scale dairy farming sector in the study area. The questionnaire was also pre-tested and all interviews were performed by the same persons. During the interviews, the questions were continuously evaluated to make sure that the farmers understood them correctly. Other biases could have arisen due to the exclusion of certain villages or households, but as only one village and five households were excluded this is not expected to influence the results. We therefore consider the results to give a representative picture of local knowledge, attitudes and practices related to brucellosis among small-scale dairy farmers in the study area.

This study showed poor knowledge of brucellosis and abundant high-risk behaviours among the farmers. Poor knowledge and high-risk behaviours strengthens the logic for including health education as part of control programmes. Studies with the aim to detect high-risk behaviours among livestock owners could prove to be valuable in order to develop cost-effective strategies that minimize the risk of exposure to *Brucella* spp. in countries where the disease is still not under control.

## Supporting Information

S1 Questionnaire Knowledge, Attitude, Practice (KAP) Study—Brucellosis in Tajikistan. (DOC)
