# Supplementary material for: A Study of Knowledge, Attitudes and Practices Relating to Brucellosis among Small-Scale Dairy Farmers in an Urban and Peri-Urban Area of Tajikistan
Source: PLoS One. 2015 Feb 10;10(2):e0117318. doi: 10.1371/journal.pone.0117318 (PMC4323107; doi:10.1371/journal.pone.0117318)
Supplement: S1 Questionnaire — (DOC) [file pone.0117318.s001.doc]

**Knowledge, Attitude, Practice (KAP) Study – Brucellosis in Tajikistan**

**Знания, Отношение, Практика (ЗОП) - Бруцеллёз в Таджикистане**

This questionnaire will take approximately 20 minutes to answer. Please, be assured that any information you provide will be anonymous and no personal information collected will appear in any documents or reports based on this survey.

Чтобы ответить на вопросы этой анкеты вам понадобится приблизительно 20 минут. Можете быть уверены, что вся информация, предоставленная вами, будет анонимной, и никакая личная информация, собранная на основе этого исследования, не появится ни в каких документах.

**Part 1 – Profiling**

**Часть 1 – Профилирование**

Village name:………………………………… Coordinates:…………………………………………

Название деревни: ………………………. Координаты:………………………………………….

Who in your family is mainly responsible for milking the dairy cows and handling the milk?

Кто из членов вашей семьи несёт основную ответственность за молочных коров?

……………………………………………………………………………………………………………………..

If someone else, ask to talk to him/her.

Если кто-то другой, попросите поговорить с ним/с ней.

Gender: Male Female

Пол: Мужчина Женщина

Number of dairy cows:…………… Number of other cattle:…………………..

Количество молочных коров : ……….... Количество крупного рогатого скота: ...............

Number of sheep:…………….. Number of goats:………………

Количество овец: ......... Количество коз: ................

If other animals, please specify:

Если есть другие животные, пожалуйста, назовите:

Species:…………………….. Number: ………………………...

Вид ……………………….. Количество ……………………..

Species:…………………….. Number: ………………………...

Вид ……………………….. Количество ……………………..

**Part 2 - Farm characteristics**

**Часть 2 - Характеристики фермы**

1. Do you sell your cattle on regular basis? Yes No

Регулярно ли вы продаёте крупный рогатый скот? Да Нет

If yes, how many per year? ………………………..

Если да, то сколько в год? .........................

If yes, where or to whom do you sell your cattle? Don´t read out.

Если да, то где или кому вы продаёте крупный рогатый скот?

Не зачитывать варианты.

Local market

Местный рынок

Market in neighboring village

Рынок в соседней деревне

Relatives/friends/neighbors

Родственники/друзья/соседи

Other people in the village

Другие люди в деревне

Others Please specify:……………………………………………….........................

Другие Пожалуйста, назовите ............................................................

1. Do you sell your sheep/goats on regular basis? Yes No

Регулярно ли вы продаёте овец/коз? Да Нет

If yes, how many per year? ………………………

Если да, то сколько в год?.........................

If yes, where or to whom do you sell your sheep/goats to? Don´t read out.

Если да, то где или кому вы продаёте овец/коз?

Не зачитывать варианты.

Local market

Местный рынок

Market in neighboring village

Рынок в соседней деревне

Relatives/friends/neighbors

Родственники/друзья/соседи

Other people in the village

Другие люди в деревне

Others Please specify:…………………………………………………………………

Другие Пожалуйста, назовите: ...................................................

3. Do you sell fresh milk or Smetana (or youghurt) on regular basis from the cattle?

Yes No

Регулярно ли вы продаёте молоко или молочные продукты крупного рогатого скота?

Да Нет

If yes, how frequently?……………………………………….

Если да, то как часто? .....................................

Who do you sell your milk/milk products to?

Кому вы продаёте молоко/молочные продукты?

Dairy milk processing plants

Молочныe комбинаты

Direct to consumers

Напрямую потребителю

Others, please specify ……………………………………………………………………………………….

……………………………………………………………………………………………………………...............

Другие. Пожалуйста, назовите ...........................................................................

..............................................................................................................................

4. Do you boil the milk or smetana before selling it? Yes No

If yes, for how long?.................................................

5. In the past year, have you received or bought a new cow/cattle?

Yes No

Покупали или получали ли вы в прошлом году новых коров или рогатый скот?

Да Нет

If yes, from where? Don´t read out.

Если да, то откуда? Не зачитывать варианты.

Local market

Местный рынок

Market in neighboring village

Рынок в соседней деревне

Relatives/friends/neighbors

Родственники/друзья/соседи

Other people in the village

Другие люди в деревне

Other Please specify:………………………………………………………………

Другие Пожалуйста, назовите ...................................................

6. Who in the household assist during calving?

Кто в семье может помочь в случае проблемы с отёлом?

Adult male(s)

Взрослый мужчина (мужчины)

Adult female(s)

Взрослая женщина (женщины)

Employee male(s)

Наёмный мужчина (мужчины)

Employee female(s)

Наёмная женщина (женщины)

Others Please specify……………………………………………………………..

Другие Пожалуйста, назовите ...................................................

7. Have you had any abortions or stillbirth among your cows during the last year?

Были ли в прошлом году аборты или мёртворожденные у ваших коров?

Yes If yes, please specify how many………………………………………..

Да Если да, то, пожалуйста, назовите сколько.......................

No

Нет

**Part 3 – Awareness of Brucellosis**

**Часть 3 - Осведомлённость о Бруцеллёзе**

1. Have you heard of the disease Brucellosis?

Слышали ли вы о заболевании Бруцеллёз?

Yes

Да

No If no, go to question 14

Нет Если нет, идите к вопросу 14

If yes, from where did you get the information?

Если да, то где вы получили эту информацию?

........................................................................................................

1. Which animals can get infected with Brucellosis?

Какие животные могут заразиться Бруцеллёзом?

………………………………………………………………………………………………………………………..

…………………………………………………………………………………………………………………………

1. Can human be infected with Brucellosis?

Может ли человек заразиться бруцеллёзом?

Yes

Да

No

Нет

If yes, what symptoms?

Если да, то какие симптомы?

……………………………………………………………………………………………………………….

1. Do you know how spread occurs between animals? Yes No

Знаете ли вы как передаётся заражение среди животных? Да Нет

…………………………………………………………………………………………………………………………

…………………………………………………………………………………………………………………………

1. Do you know how a human can be infected from an animal? Don’t read the options.

Знаете ли вы как передаётся заражение среди людей: Не предлагать варианты

Insect bites

Укусы насекомых

Milk

Молоко

Cheese

Сыр

Aborted fetuses and placentas

Выкидыши и плаценты

Offal

Требуха

Other Please specify: ……………………………………………………………………

Другие Пожалуйста, назовите ........................................................

Don´t know

Не знаю

1. Do you know if there is any national program concerning Brucellosis?

Известны ли вам какие-либо государственные программы, связанные с Буруцеллёзом?

Yes No

Да Нет

1. a. Do you know if there is any treatment for Brucellosis in cows/sheep/goats? Yes No

Знаете ли вы о каком-либо имеющемся лечении Бруцеллёза? Да Нет

If yes, what kind and for how long?............................................................................

Если да, то какое лечение и как долго? .....................................................................................................................................

……………………………………………………………………………………………………………………………….

b. Do you know if there exist any vaccination for Brucellosis in cows/sheep/goats?

Yes No

1. Have any person in the family been to the doctor and been told they have brucellosis? Yes No

Диагностировали у кого-либо в вашей семье Бруцеллёз? Да Нет

If yes, how do you believe the they got infected?.......................................................

………………………………………………………………………………………………………………………………………..

Если да, то как, по вашему мнению, произошло заражение?........................................

…………………………………………………………………………………………………………………………………………

1. Have you been told by the veterinarian that some of your sheep/goats or cows have had Brucellosis?

Диагностировали ли Бруцеллёз у какого-либо животного в вашем хозяйстве?

Yes No

Да Нет

If yes, how do you believe they got infected?.......................................................

Если да, то как, по вашему мнению, произошло заражение?.........................................

………………………………………………………………………………………………………………………………………….

1. Have your cattle been vaccinated against any disease? Yes No

Вакцинирован ли ваши коровы от каких-либо заболеваний? Да Нет

If yes, which disease(s):……………………………….…………………………………………………….

Если да, то от какого заболевания (заболеваний)?...........................................

………………………………………………………………………………………………………………………….

1. Have your sheep/goats been vaccinated against any disease? Yes No

Вакцинированы ли ваши овцы/козы от каких-либо заболеваний? Да Нет

If yes, which disease(s):……………………………….……………………………………………………..

Если да, то от какого заболевания (заболеваний)?...........................................

…………………………………………………………………………………………………………………….........

1. Who do you talk to most regularly about animal health issues?

С кем вы чаще всего говорите о проблемах здоровья у животных?

Family member/friend

Член семьи/друг

Neighbor

Сосед

Veterinarian

Ветеринар

Village chief/other community leader

Глава деревни/другой руководитель

Other Please specify……………………………………………...................................

Другой Пожалуйста, назовите...................................................................

**Part 4 – Attitudes**

**Часть 4 - Отношение**

Skip question 1 if the answer was NO on the question “have you heard of the disease Brucellosis”. (3:1)

Пропустите вопрос 1, если ответ на вопрос "Слышали ли вы о заболевании Бруцеллёз?", был НЕТ

1. Do you believe any family members are at risk of acquiring brucellosis?

Считаете ли вы, что кто-либо из членов семьи подвержен риску заболевания Бруцеллёзом?

Yes No

Да Нет

If yes, which family member(s) do you think is/are most susceptible to infection?

Если да, то кто из членов семьи, по вашему мнению, подвержен наибольшему риску заражения?

………………………………………………………………………………………………………………………………..

1. If an animal in your household get Brucellosis, how serious do you consider this to be.

Если животное в вашем хозяйстве заразилось Бруцеллёзом, насколько вы считаете это серьёзным?

Cattle: Коровы: Not serious Quite serious Very serious

Sheep/goat Овцы/козы: Not serious Quite serious Very serious

1. Do you need/would you like more information on brucellosis?

Yes No

Нуждаетесь или хотели бы вы получить больше информации о Бруцеллёзе?

Да Нет

If yes, how would you like to receive that information?........................................

Если да, то как бы вы хотели получить эту информацию?................................

…………………………………………………………………………………………………………………………….

**Part 5 - Practices**

**Часть 5 - Практика**

Skip question 6 if the answer was NO on the question “have you heard of the disease Brucellosis”.

Пропустите вопрос 6, если ответ на вопрос "Слышали ли вы о заболевании Бруцеллёз?", был НЕТ

1. How often do you wash your hands after milking the cows?

Как часто вы моете руки после доения коров?

Every time

Каждый раз

Frequently

Часто

Sometimes

Иногда

Rarely

Редко

Never

Никогда

1. If answering *sometimes, rarely, never* - why?

Если вы ответили: *иногда, редко, никогда* - почему?

Not important/necessary

Не важно/нет необходимости

No soap/not enough soap

Нет мыла/недостаточно мыла

No clean water

Нет чистой воды

Other reasons, please specify……………………………………………………………………..

Другие причины, пожалуйста, назовите……………………………………………………

1. What do you do with dead fetuses (calf, lamb, kid)?

Что вы делаете с мёртвым плодом? (телёнка, ягнёнка, козлёнка)

……………………………………………………………………………………………………………………………

……………………………………………………………………………………………………………………………

1. Do you take any specific actions to protect yourself when dealing with cows having an abortion or with placenta/dead fetuses? Don´t read out.

Принимаете ли вы какие-либо меры предосторожности, имея дело с абортировавшей коровой или с плацентой/мёртвым плодом? Не зачитывать варианты.

Use gloves

Использую перчатки

Wash hands

Мою руки

Others Please, specify………………………………………………………………..

Другое Пожалуйста, назовите…………………………………………………..

1. If you suspect an animal being sick, what do you do?

.......................................................................................................................................
........................................................................................................................................

1. If you suspect an animal having Brucellosis, what do you do? Don´t read out

Что вы будете делать, если заподозрили у животного Бруцеллёз? Не зачитывать варианты.

Report it somewhere

Сообщите куда-либо

Seek veterinary assistance

Будете искать ветеринарную помощь

Sell the animal(s)

Продадите животное (животных)

Slaughter the animal(s)

Забьёте животное (животных)

Keep the animal(s)

Сохраните животное (животных)

Treat the animal(s)

Будете лечить животное (животных)

Others Please specify…………………………………………………………………

Другое Пожалуйста, назовите…………………………………………………..

1. If you buy a new animal, do you take any actions to assure the animal is healthy? Don’t read out

Когда вы покупаете новое животное, убеждаетесь ли каким-либо образом, что оно здорово?

Не зачитывать варианты.

Yes No If yes, how?

Да Нет Если да, то как?

Trust in own experience

Доверяюсь собственному опыту

Use veterinary inspection

Пользуюсь ветеринарным осмотром

Require laboratory tests

Запрашиваю лабораторные тесты

Demand immunization certificates for Brucellosis

Требую сертификат вакцинации от Бруцеллёза

Buy from persons you trust have healthy animals

Покупаю у лиц, которым доверяю, что их животные здоровы

Others Please specify……………………

Другое Пожалуйста, назовите

……………………………………………………………………………………………………………………………………..

1. Do you drink fresh milk or smetana? Yes No

Пьёте ли вы свежее молоко? Да Нет

If no, please specify what you do with the raw milk/milk products before consumption?..............................................................................................................

Если нет, то укажите, что вы делаете с сырым молоком/молочными продуктами перед употреблением?..............................................................................................

………………………………………………………………………………………………………………………………….

1. Does the nutritive value change after boiling the milk? Yes No

Изменяются ли питательные свойства молока после кипячения? Да Нет

**Part 6 - Demographics**

**Часть 6 - Демографические данные**

1. What is the last grade of formal education you completed?

Какое последнее полученное вами образование?

No formal school Не учился/не училась в школе

Some primary Несколько начальных классов

Completed primary Полное начальное

Some secondary School Неполное среднее

Completed secondary School Полное среднее

Technical/Vocational Техническое/профессиональное

Some/Completed Pre-University Подготовительные курсы института

Some/completed Diploma Degree Незаконченное высшее

University Высшее

Post graduate Аспирантура

Don´t know Не знаю

Refused Отказываюсь отвечать

1. What is the main language spoken by people living in this household?.........................

На каком языке в основном говорят в вашей семье?

…………………………………………………………………………………………………………………………………..

1. Can you read any language?

Умеете ли вы читать на каком-либо языке?

Yes, please specify what language………………………………………………………..

Да, назовите на каком языке.................................................................

No

Нет

1. How many people live in your household (including children, relatives)?

Сколько людей проживает в вашей семье (включая детей и родственников)

Other comments?.....................................................................................................................................

Другие комментарии?.............................................................................................................................

…………………………………………………………………………………………………………………………………………………………….

..................................................................................................................................................................
